# Spatial distribution and clustering of contagious caprine pleuropneumonia in goats of Amhara region, Ethiopia

**DOI:** 10.1016/j.heliyon.2024.e38180

**Published:** 2024-09-21

**Authors:** Asres Zegeye, Wudu Temesgen Jemberu, Tsegaw Fentie, Sefinew Alemu Mekonnen, Wassie Molla

**Affiliations:** a Sirinka Agricultural Research Center, Amhara Agricultural Research Institute, Woldia, Ethiopia; b University of Gondar, College of Veterinary Medicine and Animal Sciences, Gondar, Ethiopia; c International Livestock Research Institute, P.O.Box 5689, Addis Ababa, Ethiopia

**Keywords:** Amhara region, Cluster, Contagious caprine pleuropneumonia, Goats, Spatial distribution

## Abstract

A cross-sectional study with a multistage cluster sampling technique was undertaken from January to July 2019 in Amhara region to identify spatial clusters and distributions of contagious caprine pleuropneumonia (CCPP) hotspots. In the study, a total of 2080 goats from 258 flocks across 60 villages in 12 districts were tested for CCPP serostatus using Competitive Enzyme-Linked Immunosorbent Assay (C-ELISA). Villages were taken as an aggregate unit to detect spatial distribution and clustering. Spatial autocorrelation, interpolation, and spatial scan statistics analyses were employed to analyze the spatial patterns and clusters of CCPP serostatus. The overall seroprevalence of CCPP at the animal level was 5.1 % (95 % CI: 3.8–6.6). The spatial distribution of CCPP seropositivity was non-random at the village level (Moran's I: 0.400791, P-value <0.01). Two statistically significant spatial clusters of CCPP seropositive were found: one in West Gojjam and the other encompassing parts of North Wollo, Oromiya Liyu zone, and South Wollo zones. The CCPP seropositivity showed spatial variation across the Amhara region. High CCPP seropositive spots were found in the southwestern and northeastern parts of the Amhara region. Generally, the findings of this study provide valuable insights in developing disease control strategies, prioritizing target intervention areas and measures, and designating CCPP disease-free zones. Applying movement restrictions and vaccinating goats will be important intervention measures to prevent and control CCPP in the region.

## 1 Introduction

Contagious caprine pleuropneumonia (CCPP) is a notifiable disease that causes significant economic losses in regions such as the Middle East, North and East Africa, and Asia [1]. In Ethiopia, particularly in the Amhara region, goats are vital for farmers' livelihood security. However, the productivity of goats in the country is severely constrained by various infectious diseases, with contagious caprine pleuropneumonia being a major obstacle [[2], [3], [4], [5], [6]].

Contagious caprine pleuropneumonia is a serious goat disease caused by *Mycoplasma capricolum subspecies capripneumoniae* (Mccp) [7,8]. The disease is characterized by anorexia, fever, dyspnea, polypnea, active coughing, nasal discharges, grunting, inability to move, standing with their front legs wide apart, stiff and lengthened neck, and frequent salivation [6,7]. It transmits through close contact and the inhalation of respiratory droplets from infected or carrier animals, introducing the bacteria into the flock. The disease causes high economic loss due to its higher mortality, inferior meat and milk output, diagnostic expenses, treatment costs, control costs, and disruption of trade. The morbidity and mortality rates of the disease among a susceptible population reach 100 % and 60–80 %, respectively [1,[9], [10], [11]].

A 25.7 % overall pooled prevalence of contagious caprine pleuropneumonia was reported in Ethiopia [6]. The earlier studies indicated the occurrence of the disease in six regions (Oromia, SNNPR, Afar, Tigray, Somali, and Gambella) and one city administration (Dire Dawa), with prevalence proportion varying from 4.9 % [12] in Dire Dawa to 51.8 % in Oromia [13].

Describing the spatial distribution of a particular disease is an important part of disease surveillance [14]. Additionally, quantifying the spatial association and environmental patterns of a disease provides valuable insights into its transmission and control measures [[15], [16], [17]]. Identifying geographical locations with high disease transmission using geographic information systems (GIS) and spatial statistical analysis has become critical for guiding the categorization of target intervention areas. Understanding the epidemiological distribution and status of the disease at national and regional levels is crucial for its control and prevention [18,19]. However, to our knowledge, no regional studies in Ethiopia assessing the spatial distribution and clusters of CCPP have been conducted in the past. Therefore, the main objective of this study was to enhance our understanding of the infection dynamics of CCPP by identifying spatial clusters and hotspot locations in the goat population of the Amhara region. The study provides essential information on the spatial epidemiology of CCPP in the Amhara region, Ethiopia. It will also help to prioritize intervention areas and determine future strategies for more effective CCPP control.

## 2 Materials and methods

### 2.1 Description of the study area

**Fig. 1:**
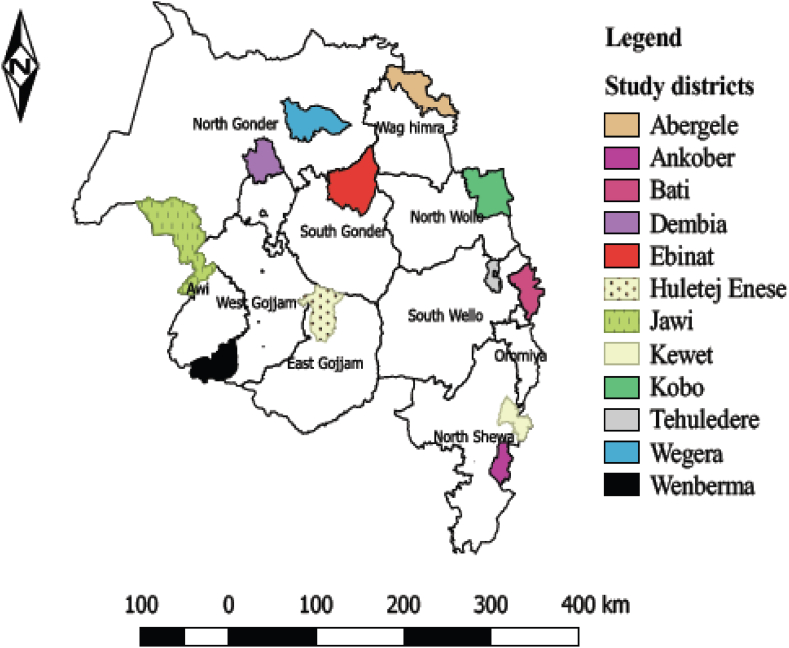
Map of study districts in Amhara Region.

The study was carried out in the Amhara regional state of Ethiopia (Fig. 1). The region has a total of about 7,766,661 goat population [20]. It is home to several goat breeds, including Abergele, Afar, Central Highland, Western Highland, and Western Lowland goats [21]. Administratively, the region is divided into 11 zones: North Gondar, South Gondar, West Gojam, Bahirdar Special Zone, Awi, East Gojam, Wag Hemra, North Wollo, South Wollo, Oromia, and North Shoa [22].

The Amhara region covers 161,828.4 km^2^ and is categorized into three agroecological zones: highlands (above 2300 m above sea level), mid-highlands (1500 to 2300 m above sea level), and lowlands (below 1500 m above sea level) [23]. The daily average temperature in the region ranges from 16 to 27 °C [24].

For this study, one or two districts were chosen from each administrative zone, excluding Bahirdar special zone The districts included in the study were Aberegele, Kobo, Tehuledere, Bati, Kewet, and Ankober in the Eastern part of the region, and Huletej Enese, Wenberma, Jawi, Ebinat, Wegera, and Dembia districts were in the Western part (Fig. 1).

### 2.2 Study animals and study design

The study subjects were goats of varying breeds, sexes and ages above six months, reared in a mixed crop-livestock production system. Because goats in the same village share common grazing and watering areas, all goats within a village were considered as a single population (epidemiological unit). To determine their CCPP serostatus, a cross-sectional study design was employed between January and June 2019.

### 2.3 Sampling methods

A multistage cluster sampling approach was used to select the study subjects. First study districts were chosen purposefully representing each zone of the region and taking into account factors such as availability of goat population and a history of not receiving the CCPP vaccination. From each selected district, one or two kebeles (smallest administrative unit) (depending the size of the district) were chosen using simple random sampling after excluding inaccessible kebeles. Using simple random sampling, two to four villages were chosen from each selected kebele (the smallest administrative unit), based on the total number of villages within each kebele. Again, from each selected village, goat flocks (goats owned by a farmer) were selected using simple random sampling, and finally individual goats were selected using systematic random sampling from the selected villages. For this study, a total of 258 flocks from 12 districts, 24 kebeles, and 60 villages were included. The lists of kebeles were found from the agricultural office of each district, while lists of villages and goat flocks within those kebeles were obtained from the corresponding kebele administrative offices.

### **2.4** Sample size determination

The sample size for this study was calculated using the cluster sampling method [25,26]. The sample size required to estimate the prevalence with a defined level of precision was calculated as follows:

$$n=gc=\frac{P\left(100-P\right)D}{{SE}^{2}}$$

Where, **n** = the sample size, **p** = the prevalence as a percentage, **D** = the design effect, SE = is the precision (standard error), g = the average number of individuals sampled per cluster, and, **C** = the number of clusters (villages) sampled. The next formula gives the design effect:

D=1+(g−1)ICC

Where, intracluster correlation coefficient (ICC) is a measure of the relatedness of clustered data. Otte and Gumm [27] reported that estimate of ICC for most infectious diseases does not exceed 0.20. By sampling about 34 animals per village, with an estimated CCPP prevalence of 25.7 % in Ethiopia [6] and a desired precision of 2.7 % gave around 60 clusters (villages). This gives an overall sample size of nearly 2006 animals. Finally, we managed to sample 2080 individual goats. The clusters and the overall sample size were distributed proportionally across 12 study districts and 24 kebeles.

### 2.5 Data collection procedure

Blood samples were collected from apparently healthy goats using sterile disposable needles and plain vacutainer tubes. About 6 ml of blood was drawn from the jugular vein of each non-vaccinated animal and allowed to clot at room temperature in a skewed position. Centrifugation was done to separate the serum from the clotted blood. It was shifted and stored at −20 °C until shipping for testing. Finally, the serum samples were sent in ice packs to the National Veterinary Institute (NVI) at Debre Ziet, Ethiopia, for CCPP antibody testing. Additionally, data regarding the locations of sampled areas (zones, districts, kebeles, and villages), household names, and global positioning system coordinates (longitude, latitude, and altitude) were collected for spatial analysis.

#### 2.5.1 Laboratory analysis of serum sample

The serum samples were investigated using IDEXX CCPP competitive enzyme-linked immunosorbent assay (C-ELISA). The method involves mixing serum samples with an antibody detection solution in plates and then shifting it into covered microplates. In the detection solution, antibodies in the serum sample form an immunological reaction with the McCP antigen by competing with the monoclonal antibody (mAb) for specific epitopes. Unreacted material was washed away, and an anti-mouse antibody enzyme was added.

In the presence of an immunological reaction between Mccp antigen and antibodies, Mab binding was withdrawn. However, in the absence of Mccp antibodies in the test sample, the Mab can bind to a specific epitope. The unbound conjugate was washed away, and an enzyme substrate was added. In the presence of an enzyme, the substrate reacts and gives a blue color. The succeeding color change is inversely proportional to the amount of anti-Mccp antibodies. According to the company's method, the result was expressed as "percent inhibition" by comparing the optical density of the test well with that of the Mab control wells [28]. Results were calculated by percent inhibition, $PI=\frac{OD\;Mab-OD\;test}{OD\;Mab-OD\;CC}*100$.Where, OD = Optical density at 450 nm, Mab = Monoclonal antibody, CC = conjugate concentrate. An inhibition value of 55 % was used as a cut-off between positive and negative samples.

### **2.6** Data management and analysis

#### 2.6.1 Animal-level and flock-level prevalence

The apparent prevalence at the animal and flock levels was calculated using the following formula:. $Animal\;level\;Apparent\;prevalence=\frac{number\;of\;positive\;sample}{numbe\;of\;total\;sampled\;population}*100$.

$$Flock\;level\;Apparent\;prevalence=\frac{{number\;of\;positive\;flocks}^{\#}}{total\;numbe\;of\;sampled\;flocks\;}*100$$

∗if one animal from a flock tests positive, the entire flock is considered positive∗

#### 2.6.2 Spatial autocorrelation analysis and disease mapping

During data analysis, a village was taken as the unit of analysis for this study. Spatial autocorrelation analysis was employed to evaluate whether disease distribution patterns were dispersed, clustered, or random in the study area. Moran's I was used to test spatial dependency between cases of the diseases. The Z-score test was used to assess the significance of the estimate for Moran's I. A statistically significant Moran's I (p < 0.05) was used to reject the null hypothesis of no spatial dependency and indicate the presence of spatial autocorrelation [29]. ArcGIS version 10.6 was employed to conduct spatial autocorrelation analysis.

The disease prevalence across the region was mapped through spatial interpolation of the disease prevalence data collected from sampled villages. Kriging spatial interpolation method was used to predict the prevalence of CCPP in the unsampled neighboring villages of the Amhara region. The predictions were made at the zonal level.

#### 2.6.3 Hot spot disease cluster detection

Hot spot disease cluster detection was done using spatial scan statistics. The spatial scan statistical analysis was conducted by using SaTScan software version 9.6 [30]. SaTScan was employed to pinpoint statistically significant spatial clusters of cases by progressively scanning windows across the study areas. Statistical significance cluster of cases was evaluated using the Bernoulli model. Villages were taken as the unit of analysis to identify hotspot areas and clusters during analysis, as goats managed within the same village shared watering and grazing areas, and were considered as one epidemiological unit. During the analysis, number of animals within each village that were tested seropositive for CCPP were considered as cases, while those animals tested negative in each village were considered as controls. In the analysis, the ratio of cases and controls in and out of a circular scanning window was compared. The default highest spatial cluster size of 50 % of the population was taken as an higher limit for a population at risk, allowing both small and big clusters to be identified [31]. The log-likelihood ratio test statistics were produced by Monte-Carlo simulation and were used to compare the number of observed cases within the scanning window to the expected number of cases in this window. Observed cases represent the actual number of animal cases recorded in a specific geographic area, while expected cases denote the number of animal cases anticipated to occur in the same geographic area based on a reference population. The circle with the maximum likelihood ratio, which contained more cases than expected was recognized as the most probable (primary) cluster, suggesting it is less likely to have occurred by chance. A significance level of alpha <0.05 was taken to determine whether the cluster was statistically significant [32]. From SaTScan output, hotspot areas were identified based on the value of relative risk (RR). An area with a relative risk value (RR) ≥ 1.00 and statistically significant (P ≤ 0.05) and categorized as a hotspot [33]. The output of the analysis was projected onto ArcGIS version 10.6.

## 3 Results

### 3.1 Spatial distribution of contagious caprine pleuropneumonia in Amhara region

**Fig. 2:**
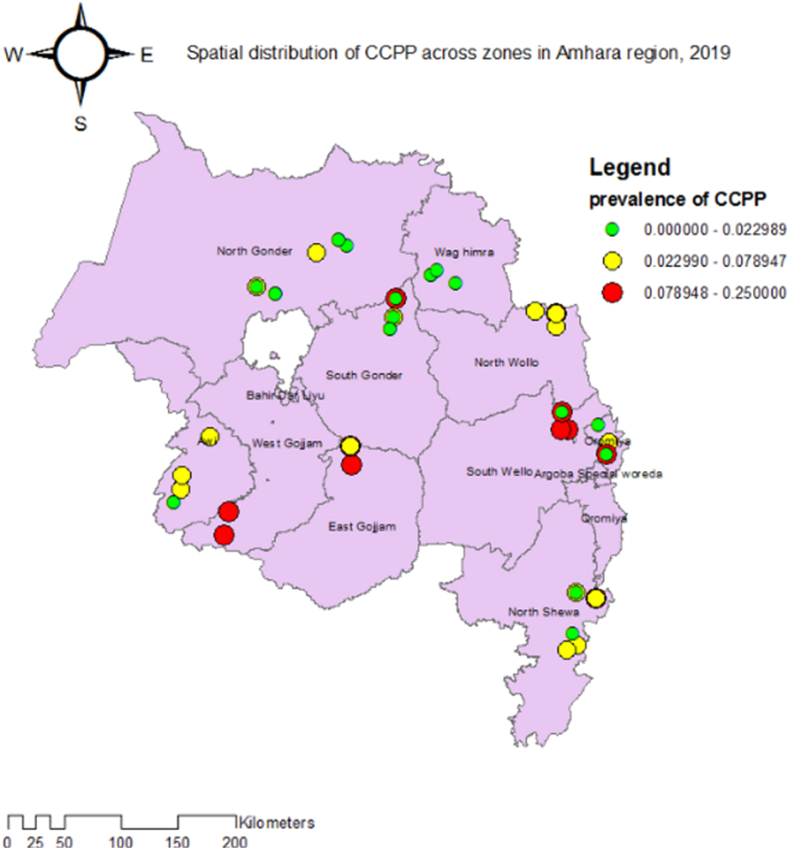
Spatial distribution of contagious caprine pleuropneumonia village-level clusters across Amhara region, 2019.

The animal-level seroprevalence of CCPP in the study was 5.1 % (95 % CI: 3.8–6.6). The distribution of CCPP prevalence at the village level is shown in Fig. 2. Geographically, a high level of CCPP seroprevalence (about 7.89–25.0 %) was observed in a few villages of South Gondar, East Gojam, South Wollo, West Gojam, and Oromia Liyu zones of the Amhara region. In contrast, a low level of CCPP seroprevalence (0.0%–2.2 %) was observed in most villages of North Gondar, South Gondar, Waghimira, North Shoa, and Oromia Liyu zone (Fig. 2).

### 3.2 Spatial autocorrelation

The Moran's I value of the spatial autocorrelation analysis was found to be 0.4. This value of Moran's I was statistically significant (P = 0.001), indicating the presence of positive spatial autocorrelation for CCPP serostatus among neighboring villages in the sampled area.

### 3.3 Spatial interpolation of contagious caprine pleuropneumonia serostatus among goats of Amhara region

**Fig. 3:**
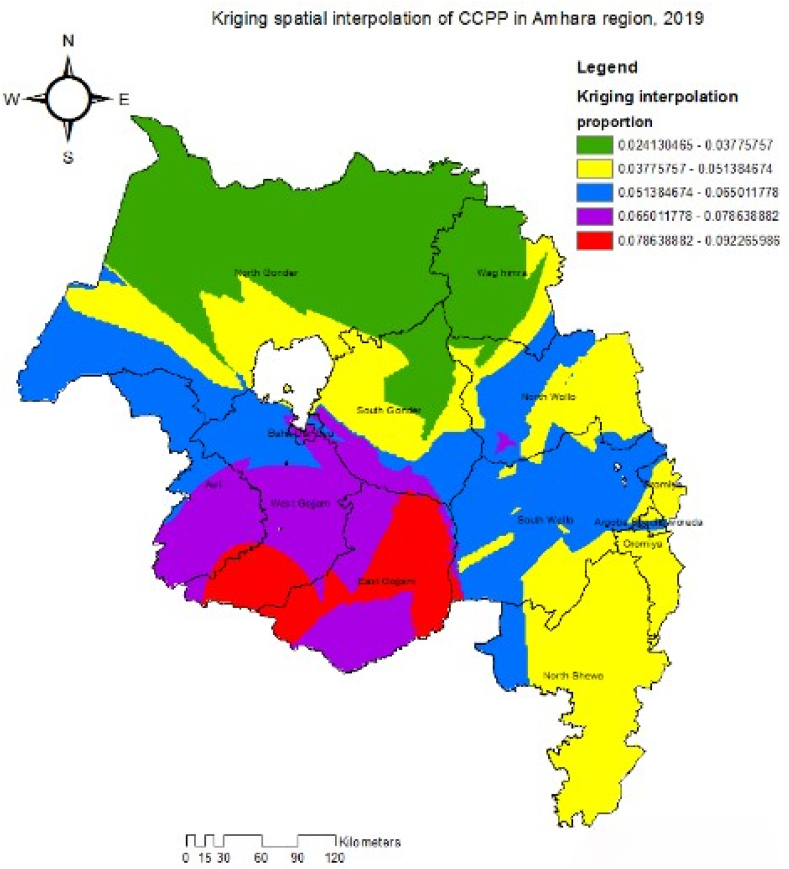
Kriging spatial interpolated prevalence of CCPP in goats across zones of Amhara region, 2019.

The spatial interpolation indicated a low CCPP seropositivity in the North and a high CCPP seropositivity in the West of the region (Fig. 3). The red gradient on the map in Fig. 3 highlights the areas predicted to have the highest CCPP seropositivity (7.9%–9.2 %) in some parts of the East and West Gojam zones. Whereas, the green ramp color indicates low CCPP seropositive areas (2.4–3.7 %) in North Gondar and Waghimira zones.

### 3.4 Spatial clustering and hot spot of contagious caprine pleuropneumonia in Amhara region

**Fig. 4:**
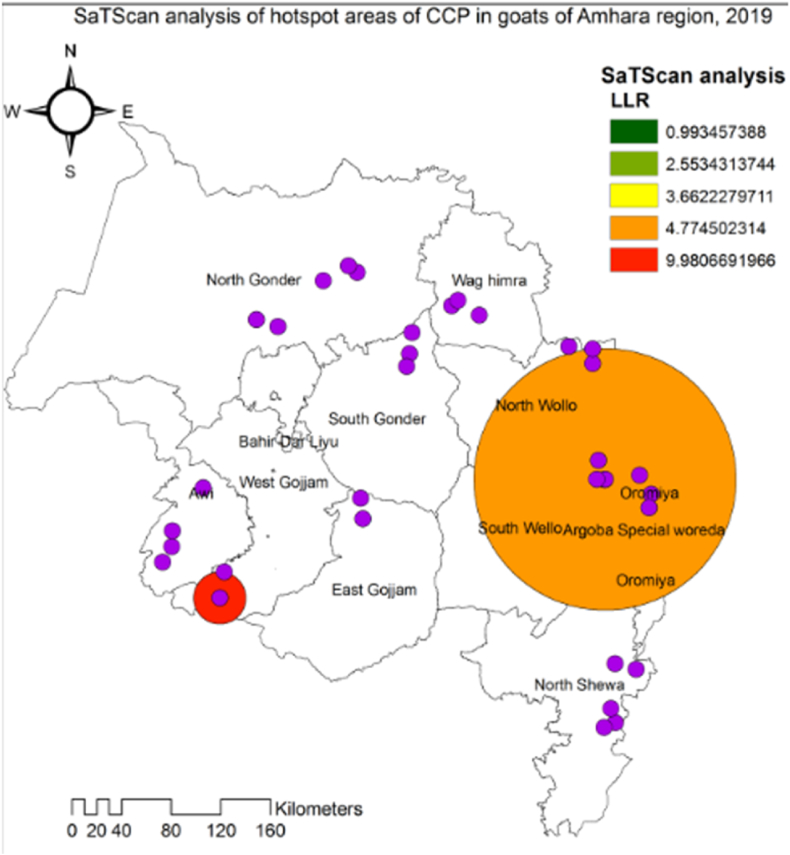
Geographic map of hot spot clusters of CCPP serostatus in goats aggregated at village level across zones of Amhara region, 2019.

The results of SaTScan cluster analysis revealed the spatial cluster and hot spot of CCPP in Amhara region (Table 1 and Fig. 4). Five spatial clusters of CCPP were detected across the study areas, but only two of these clusters were statistically significant (P < 0.05). The CCPP clusters involved 2 to 15 villages and 67 to 499 goats (Table 1).

**Table 1:** CCPP spatial clusters by study districts in Amhara region, 2019.

**Clusters**	**Districts**	**Coordinates/radius (km)**	**Number villages**	**No. of goats**	**Observed cases**	**Expected cases**	**Observed/Expected**	**Relative risk**	**P-value**
Primary (Most likely)	Weberima	10.442000 N, 36.879000 E/21.04 km	3	102	17	5.15	3.30	3.75	0.000
2nd secondary	Tehuledere, Kobo, Bati, Abergele and Ankober	11.310000 N, 39.720000 E/105.59 km	15	499	39	25.19	1.55	1.87	0.048
3rd secondary	Tehuledere	11.310000 N, 39.720000 E/6.65 km	2	91	11	4.59	2.39	2.56	0.183
4th secondary	Kobo	12.256000 N, 39.629000 E/4.1 km	2	67	8	3.38	2.37	2.48	0.536
5th secondary	Bati	11.102000 N, 40.043000 E/5.3 km	4	80	7	4.04	1.73	1.79	0.993

Relative Risk/Risk Ratio (RR): the ratio of an estimated risk within the cluster and an estimated risk outside the cluster, Significant at p-value˂0.05.

In the primary cluster, there were 3 villages located within a radius of 21.04 km, with 17 observed cases and an expected number of 5.15 cases. In the second-secondary cluster, there were 15 villages with a radius of 105.59 km. The observed cases were 39, the expected cases were 25.19. Across all clusters, the ratio of observed to expected cases ranged from 1.55 to 3.30 (Table 1).

The primary cluster (hotspot) area of CCPP, indicated by a bright red ring in Fig. 4, was located in the West Gojam zone. The second hotspot area, marked by the orange window in Fig. 4, was found in the North Wollo, South Wollo, and Oromiya Liyu zones.

## 4 Discussion

This study identified hotspot areas, spatial clusters, and distribution of CCPP in goats within the Amhara region of Ethiopia. This information will aid in controlling CCPP in the region by helping to identify and prioritize target intervention areas.

The study indicates the presence of positive spatial autocorrelation, as evidenced by clustering with a significant positive Moran's I value. It suggests that the seropositivity of goats in neighboring villages tends to be similar. This suggests that the spread of the disease is affected by local factors, including contacts between infected and susceptible animals, environmental conditions, and other regional factors [34]. Additionally, areas with high CCPP seroprevalence were found to be in close proximity and exhibited clustering distribution patterns. From an epidemiological perspective, this indicates the infectious and contagious nature of the disease [35]. Moran's I serves as a powerful and robust tool for detecting patterns in disease distribution [36]. Spatial autocorrelation analysis is an important method for evaluating the existence of spatial clustering. In addition, this allows for understanding the spatial structure and detecting spatial dependence [37].

The spatial interpolation in this study revealed the highest seropositivity in villages located in the East and West Gojam zones and the lowest seropositivity in north Gondar, some parts of south Gondar, and Waghimira zones. Space-based predictions on the risk of infection are utilized for disease control strategies in areas considered to be at a high risk of infection [38]. In underdeveloped countries, geographical information system-based models provide valuable information for disease prediction and facilitate disease control programs for livestock experts [39,40].

The spatial point prevalence analyses conducted in this study revealed higher CCPP seroprevalence in villages located in West Gojam, South Gondar, East Gojam, South Wollo, and the Oromiya Liyu zones (ranging from 7.89 % to 25.00 %). The high prevalence among goats within these villages indicates active circulation of the CCPP agent and suggests that the management practices in these areas might contribute to the spread of the disease. This could be associated with common practices such as free animal movement, communal grazing, and watering areas. These practices facilitate the transmission of the disease within and between villages [41]. There is also a report that suggests that disease dynamics can vary between different areas, potentially due to variations in management practices and other environmental factors [42].

The results of the spatial scan statistical analyses confirmed that the point prevalence of CCPP was clustered in villages within the Amhara region. This spatial clustering indicates the contagious and infectious nature of CCPP [43]. Out of the five clusters analyzed, two were statistically significant spatial clusters (p < 0.05). The most probable spatial clusters of CCPP prevalence was identified in the Western Gojam zone of Western Amhara. This could be attributed to the ongoing informal trade of goats from Western Wellega and the movement of transhumance goat farmers from Benishangual Gumiz and Western Wellega to the Weberima district in the West Gojam zone [44]. Infectious disease clusters in small ruminant may occur when goats within a village share common risk factors [45] or when infected animals are moving freely from one village to another [46].

The results of the hotspot analysis also revealed that high-risk areas were clustered in the western and eastern parts of the region, specifically in the Western Gojam zone, North Wollo zone, Oromiya Liyu zone, and Southern Wollo zone. The primary hotspot cluster was identified in the Weberima district, while the secondary hotspot cluster was located in Tehuledere, Kobo, Bati, and Ankober districts. These hotspot clusters might occur because these districts border other regional states of Ethiopia known for their CCPP endemic status. This is supported by other authors [47], where border regions are particularly associated with high risk due to intense and informal animal trade. Identifying spatial hotspot areas can assist policymakers in developing animal disease control, eradication, and prevention strategies at national, regional, district, and farm levels. Additionally, hotspot analysis provides valuable information for allocating resources to specific locations [48].

Generally, the findings of this study enable us to enhance the understanding of CCPP in goats of Amhara region. Besides, it also provides valuable insights for targeted interventions such as vaccination campaigns, disease surveillance, and resource allocation to prevent and control CCPP in the specific area. However, the study did not take into account some factors that could influence disease patterns, such as population density, animal movement, environmental factors, and concurrent infections. Failure to adequately account of these factors can limit the study to identify causal relationships. As a serological test was employed to establish the CCPP status in the study area, the study may provide information about the history of the disease but does not necessarily indicate the presence of the disease. Moreover, as the study was cross-sectional, it was unable to track the spread of the disease over time.

## 5 Conclusions and recommendations

Amhara region, CCPP seropositivity has spatial variation across the region. The spatial distribution of CCPP seropositivity was non-random at the village level, which indicates the presence of spatial clustering of the disease in the study area and spatial dependency between cases of the disease. Geographical differences have been observed in seropositivity of CCPP in different zones of the region. The predicted CCPP seropositivity varied from low in the north to high in the western part of the region. Western Gojam, North Wollo, South Wollo, and Oromia Liyu zones were identified as high-risk areas of CCPP. The identified clusters should be targeted when formulating strategies for CCPP control in the region.

## Data availability statement

Data used for the analysis was included as supplementary material and could be available in the manuscript.

## Ethics statement

All study procedures and animal care followed Federation of Animal Science Societies (FASS) [49] guidelines and were approved by Animal Use and Care Committee of University of Gondar.

## CRediT authorship contribution statement

**Asres Zegeye:** Writing – review & editing, Writing – original draft, Software, Investigation, Formal analysis. **Wudu Temesgen Jemberu:** Writing – review & editing, Validation. **Tsegaw Fentie:** Writing – review & editing, Validation, Supervision, Methodology. **Sefinew Alemu Mekonnen:** Software, Methodology. **Wassie Molla:** Software, Methodology, Conceptualization.

## Declaration of competing interest

The authors declare that they have no known competing financial interests or personal relationships that could have appeared to influence the work reported in this paper.
